# Characterization and pathogenicity of very virulent IBDV

**DOI:** 10.3389/fvets.2025.1670204

**Published:** 2025-09-26

**Authors:** Qi Liu, Jiangjiang Nie, Ying Hu, Qinghua Zeng, Ke Wang, Xiangdong Wu, Zheng Chen, Huali Xie, Huansheng Wu

**Affiliations:** ^1^Department of Veterinary Preventive Medicine, College of Animal Science and Technology, Jiangxi Agricultural University, Nanchang, China; ^2^Jiangxi Provincial Key Laboratory for Animal Health, College of Animal Science and Technology, Jiangxi Agricultural University, Nanchang, China

**Keywords:** infectious bursal disease virus, sequencing, very virulent, phylogenetic, pathogenicity

## 1 Introduction

Infectious bursal virus belongs to the family *Birnaviridae* and the genus *Avibirnavirus* (1). The *Birnaviridae* family has three virus genera, including *Avibirnavirus* represented by infectious bursal disease virus (IBDV), *Aquabirnavirus* represented by infectious pancreatic necrosis virus (IPNV), and *Entomobirnavirus* represented by Drosophila X virus (DXV) (2). The IBDV genome contains double-stranded RNA (dsRNA) in segments A and B. The non-structural viral protein VP5 and polyprotein VP2-VP4-VP3 are encoded in segment A (approximately 3.2 kb). Among these, the polyprotein undergoes further self-cleavage to produce the precursors VP2, VP3, and VP4. After additional processing, PVP2 yields mature VP2 and a number of tiny peptides (3). VP2 is the outer capsid protein that includes the major protective antigen and neutralizing epitopes (1). In addition, the highly variable region (HVR) of VP2 plays an important role in the cytotropism, virulence, and antigenic variation of the virus. The antigenic variation of IBDV mainly stems from the mutation in the high-variability region of VP2 (amino acids 206–350) (4). Variant strains, such as the Del-E strain in the United States, have significant differences from traditional strains in this region, causing them to evade the immune protection induced by traditional vaccines (5). Molecular epidemiological studies have shown that the VP2 of vvIBDV is highly conserved worldwide (>96% similarity), suggesting that these strains may originate from a single ancestral virus (6). It is worth noting that naturally occurring recombination events (recombination of segments A and B) have also led to the emergence of new virus strains, such as the A2dB1 recombinant strain discovered in China in recent years (7). VP3, the “scaffold protein” of the virus, not only participates in capsid assembly but also binds with the viral dsRNA to form the virus replication complex (RPC). Studies have shown that VP3 plays an important role in inhibiting the host's innate immune response (8–10). It can block the activation of interferon regulatory factor 7 (IRF7), resulting in to inhibition the type I interferon generation (11). As an RNA polymerase, VP1 has a unique “genomic ligation” activity and is capable of covalently ligating viral RNA *in vitro*. This characteristic is quite unique in the *Birnaviridae* family (12, 13). IBDV mainly targets the Bursa of Fabricius (BF), the central immune organ of young chickens, especially disrupting the IgM+ B cell subset of immature B lymphocytes. IBDV infection leads to the massive apoptosis of these cells, resulting in damaged immunosuppression (14).

They are classified into two subgroups of IBDV (type I and type II), but only serum type I causes significant damage to young chickens. Serum type I viruses can be further classified into classic strains (such as STC and F52/70), variant strains (such as Del-E and GLS), and very virulent strains (vvIBDV, such as HK46 and UK661) (6). These strains exhibit significant differences in pathogenicity, antigenicity, and molecular characteristics. Since the emergence of very virulent strains in the late 1980s, they have become a major threat to the global chicken farming industry, with a mortality rate of 70%−100% (15). IBDV demonstrates significant stability and is resistant to a variety of physical and chemical factors (16). The virus remains stable within the pH range of 2–12; is insensitive to ether, chloroform, and trypsin; and can still remain infectious after being treated at 56 °C for 5 h. This strong environmental resistance enables the virus to survive for a long time in the breeding environment, increasing the difficulty of prevention and control (17). In recent years, IBDVs isolated from chicken farms in Jiangxi Province are mainly composed of vvIBDVs and novel variant strains. For instance, a case of co-infection with vvIBDV and chicken infectious anemia virus (CIAV) was identified in a layer farm in Jiangxi in 2024. Sequencing results revealed that this vvIBDV belongs to a novel variant strain, and its genomic characteristics are significantly different from those of traditional strains. In addition, the A3B3 genotype (e.g., HLJ0504-like strain), which is prevalent nationwide, also poses a potential transmission risk in Jiangxi Province. This type of strain causes severe damage to the BF, often leading to high mortality and immunosuppression in infected chickens.

In this study, BF tissues of chickens infected with IBDV in Jiangxi Province and vaccinated with IBDV were collected. Chicken embryo inoculation was carried out, and a vvIBDV strain was isolated and identified. Both segments A and B were assembled from vvIBDV, with the genotype A3B3 (18, 19). Moreover, the pathogenicity of the SM01 strain was evaluated by infecting SPF chickens, and the results suggested that the vvIBDV strain SM01 could cause severe pathological damage to the BF.

## 2 Materials and methods

### 2.1 Chickens and embryos

Specific pathogen-free (SPF) chickens were supplied by Nanchang Miaowang Hatchery (Nanchang, China). SPF embryos were purchased from Shandong Haotai Experimental Animal Breeding Co., Ltd. (Shandong, China).

### 2.2 Sample collection

In September 2023, the 5-week-old vaccinated chickens grew slowly with poor uniformity, disheveled feathers, and severe bursal atrophy. In addition, the primary clinical concern was immunosuppression. This farm has been immunized with an attenuated IBDV vaccine. Five BF tissues were collected from diseased chickens for RT-PCR detection. The positive samples were homogenized in Phosphate-Buffered Saline (PBS), and then frozen and thawed three to five times to release the virus (frozen at −80 °C and thawed at room temperature). Subsequently, the samples were subjected to centrifugation at 12,000 g for 10 min to get the supernatants for virus isolation.

### 2.3 IBDV molecular detection

The total RNA of the BF tissues was extracted using the TRIzol reagent based on the manufacturer's instructions. The positive IBDV samples were detected according to a previously reported RT-PCR assay (20).

### 2.4 Virus isolation and genome sequencing

The suspensions of IBDV-positive BF tissues were filtered through 0.22 μm syringe filters and inoculated into 10-day-old SPF chicken embryos for viral passaging. The stained embryos were further analyzed by RT-PCR to confirm IBDV infection. To detect the purity of the isolated IBDV, avian leucosis virus (ALV), Marek's disease virus (MDV), chicken anemia virus (CAV), and fowl adenovirus serotype 4 (FAdV4) were detected by PCR; avian influenza virus (AIV) and Newcastle disease virus (NDV) were examined by hemagglutination assay. The full length of the viral genome of segments A and B was amplified by RT-PCR using the following primers, IBDVAF: GGATACGATCGGTCTGACCC, IBDVAR:CCGGACCCGCGAACGGAT; IBDVBF:GGATACGATGGGTCTGACCCTCT, and IBDVBR: GGGGGCCCCCGCAGGCGAA. The PCR products were sub-cloned into pMD19-T vectors for the next sequencing.

### 2.5 Multiple alignment and phylogenetic analysis

The segments A and B of the SM01 strain used in this study have been registered in GenBank (PQ562378.1 and PQ562379). The multi-sequence alignment was performed using Jalview software to align the nucleotide sequences with multiple referenced IBDV strains. The adjacent method of 1,000 bootstrap repetitions in MEGA 7 software was used to build the phylogenetic tree.

### 2.6 Pathogenicity evaluation

Passage was performed via the allantoic cavity inoculation method. Specifically, 9- to 11-day-old chicken embryos with a good developmental status were selected. The boundaries of the air cell and the embryo position were marked under an egg candler. A mark was made at the injection site, approximately 1 mm from the edge of the junction between the embryo surface and the air cell, avoiding blood vessels. Subsequently, the injection site was disinfected with alcohol, and a small hole of about 2 mm was created using an egg drill. Using a 1 ml syringe, 0.1–0.2 ml of virus inoculum was injected into the allantoic cavity. Thereafter, the small hole was sealed with medical tape or paraffin. The chicken embryos were then placed on an egg rack with the air cells facing upward and incubated in an incubator at 33–35 °C for 48–72 h. During the incubation period, egg candling was conducted daily to check the embryos. The death of chicken embryos was determined based on the condition of blood vessels, fetal movement, and the developmental boundary of the chorioallantoic membrane.

The SM01 strain was inoculated into 1-day-old SPF chickens through eye and nasal drops at a dose of 0.4 ml per chicken with a virus titer of 10^−5.5^ EID_50_/0.4 ml, totaling 18 chickens. Meanwhile, 18 experimental chickens of the same batch were inoculated with normal saline through the same route as the control group, with 0.4 ml per chicken. The symptoms of the test chickens were observed on a daily basis until the 14th day. After 3, 5, 7, and 14 days of virus exposure, three chickens were randomly picked from each group for weighing. Then, the BF tissues were dissected and examined. The BF tissues were weighed, and the ratio of chickens [BF (mg)/chicken (g)] was calculated. And analyze the ratio of bursa of Fabricius tissue weight to body weight, BBIX = the ratio of bursal in infected chickens/the average ratio of bursal in the control chickens (when the bursae index BBIX < 0.7, bursae are judged as atrophied) (21).

### 2.7 Histopathology analysis

Among the tissues taken on 3, 5, 7, and 14 days post-challenge, BF tissues with obvious lesions were fixed with 4% paraformaldehyde tissue fixative to make pathological sections for histological observation.

### 2.8 Statistical analysis

All statistical analyses were conducted using GraphPad Prism 9.5 (San Diego, CA, USA). *P*-value of < 0.01(^**^) was considered extremely significant.

The HE staining method consists of hematoxylin (an alkaline stain) and eosin (an acidic stain). Hematoxylin can stain the basophilic structures of tissues—such as ribosomes, cell nuclei, and ribonucleic acid in the cytoplasm—into a blue-purple color. Eosin, as an acidic stain, can stain the eosinophilic structures of tissues—such as intracellular and intercellular proteins, and most parts of the cytoplasm—into a pink color.

## 3 Descriptive results

**Figure 1:**
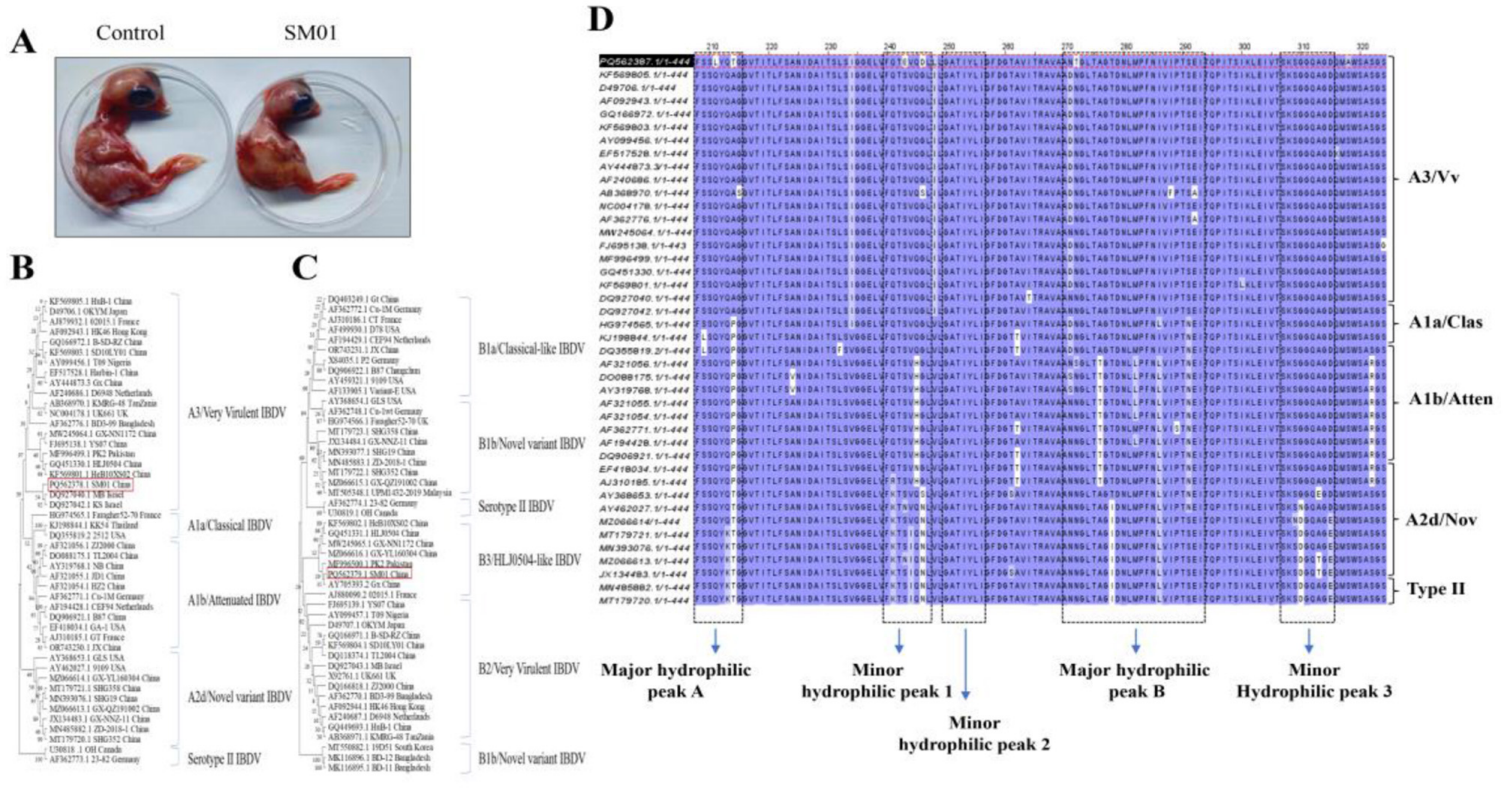
Virus isolation, phylogenetic tree construction, and sequence analysis. **(A)** Ten-day specific pathogen-free (SPF) chicken embryos were injected with the positive samples. Gradual growth of dead chicken embryos could be observed. **(B, C)** The trees of segments A and B were constructed by the neighbor-joining method with MEGA7 software. The very virulent strain isolated in this study [infectious bursal disease virus (IBDV) SM01 China] was highlighted with a black frame. **(D)** The highly variable region of SM01 VP2 was compared to those of other IBDV strains that were cited. Several significant domains are indicated by the black frames.

Total RNA was extracted from BF tissues suspected of being infected with IBDV. One of five BF tissues was IBDV positive. In addition, no other infectious viruses were present as determined by PCR. The BF homogenates were injected into 10-day SPF chicken embryos for viral passaging. After the virus of the BF tissues on the chicken embryos for three generations, it caused the death of the chicken embryos within 5 days. The alluvial membranes of the diseased and dead chicken embryos from generations 1 to 3 were hemorrhagic. Visually, white pimples could be seen on the alluvial membranes. The bleeding from the chicken embryos was severe, especially on the head, wings, and legs (Figure 1A). The embryos and their allantoic fluid were recovered for RT-PCR testing, and the results were positive (data not shown). This isolated IBDV was named the SM01 strain. Subsequently, PCR amplification of the SM01 genome was performed using the amplification primers of segments A and B, and the target gene fragments of sizes approximately 3,200 bp and 2,800 bp were expected to be obtained. The electrophoresis results showed that the size of the target fragment was consistent with the expected size, and the target band was single without miscellaneous bands (data not shown). Taken together, the IBDV strain SM01 was successfully isolated from clinically positive samples derived from a vaccinated poultry farm in Jiangxi province. The full length of segments A and B of SM01 from deceased embryos was determined and sequenced. This analysis revealed that segment A consisted of 3,259 nucleotides, while segment B consisted of 2,843 nucleotides. Segment A consists of a small Open Reading Frame (ORF) that encodes VP5 protein, and a larger ORF that encodes the polyprotein. In addition, segment B contains only one ORF that encodes VP1, the polymerase protein of IBDV.

Based on the phylogenetic trees of nucleotide sequences of polyprotein, IBDV was classified into four genogroups: A1a (classic IBDV), A1b (attenuated IBDV), A2d (novel variant IBDV), and A3 (vvIBDV) (18). The serotype II of IBDV, which is non-pathological to chickens, forms a distinguished branch (22, 23). The IBDV SM01 strain was clustered with the MB Israel strain, which is a representative strain of vvIBDV (Figure 1B). Compared with the international standard A3B3 genotype reference strain MB Israel, the SM01 strain shares a full-length nucleotide homology of 96.2%−98.7% and an amino acid homology of 98.8%−99.4% in segment A with it. The characteristic amino acids in the hypervariable region (HVR) of VP2 (e.g., 222A, 256I, and 294I) are completely consistent, which further supports its very virulent (vv) phenotype. In addition, three subgroups, B1, B2, and B3, were identified according to the phylogenetic tree of nucleotide sequences of VP1 (19). In the segment B phylogenetic tree, vv strain such as HLJ0504, classic strain such as Gt, novel variant strain such as Cu-1wt, and serotype II reference strain jointly forms a large branch. Among them, the SM01 strain forms an independent small branch with vv strains such as PK2 and Gx, and has the closest genetic relationship (Figure 1C). Compared with the domestic A3B3 genotype strain Gx, the SM01 strain exhibits a nucleotide homology of 98.2% and an amino acid homology of 99.5% with the Gx strain in segment B. The two strains exhibit high consistency in the conserved functional domains of VP1, suggesting a similarity in their replication mechanisms. Compared with the PK2 strain, the SM01 strain shares a nucleotide homology of 97.5% and an amino acid homology of 98.9% with the PK2 strain in segment B. The two strains form a tight clade in the phylogenetic tree of VP1, indicating a close evolutionary relationship between their polymerase genes. Previous reports have revealed that the HVR, amino acids 202–350 of VP2 is a crucial domain, which consists of most of the epitopes and correlates with the virulence and variation of IBDV (24). Sequence comparison of the HVR of VP2 revealed that the HVR of VP2 in the isolate SM01 has the same heptapeptide region amino acid sequence “SWSASGS” as vvIBDV, and also has vvIBDV characteristic amino acids. This specifically includes combinations of amino acid positions such as 222A, 256I, 279D, 284A, 294I, and 299S. Based on the results of genetic evolution and homology analysis, it is speculated that the isolated strain SM01 is a virulent strain of IBDV (Figure 1D).

**Figure 2:**
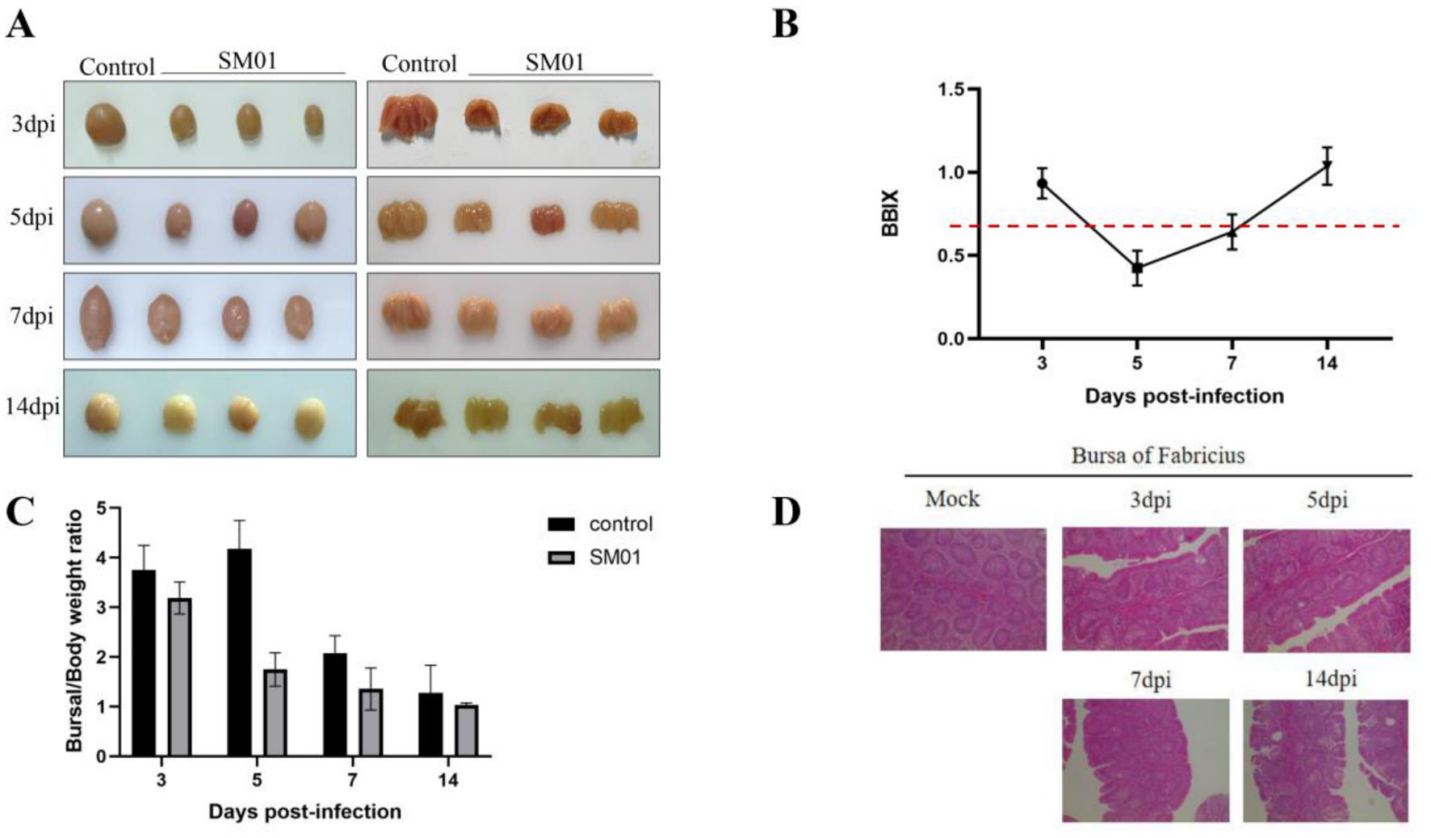
Pathological analysis of the IBDV SM01 strain. This figure provides the results of SPF chickens challenged with the IBDV SM01 strain. **(A)** Post-mortem examination of Bursa of Fabricius (BF) tissues was conducted on chickens infected with the SM01 strain. **(B)** Bursa-body index (BBIX) of chickens was evaluated at various time points following exposure to the SM01 strain. The BBIX index is lower than 0.7, indicating the severe disruption of BF. **(C)** Bursal weight ratio of challenged and control chickens was also analyzed after 3, 5, 7, and 14 days of SM01 strain infection. **(D)** The pathological damage to BF tissues was tested by HE staining after 3, 5, 7, and 14 days of infection with the SM01 strain.

The SM01 strain was inoculated into 1-day-old SPF chickens by nasal drops. As anticipated, during the animal trial, no obvious clinical symptoms were observed in the early stage of challenge (1–2 days). From day 3 onward, chickens exhibited symptoms such as depression and disheveled feathers, and the BF began to show atrophy and lymphocyte necrosis. Though none of the hens perished during the experiment, the SM01-inoculated chickens showed clinical indications of atypical IBD, such as depressed, disheveled feathers, decreased appetite and drinking, and diarrhea. All of the SM01-inoculated hens displayed bursal atrophy at the start of the 3-day post-infection period, but no overt gelatin-like deposits were seen (Figure 2A). BF tissues were collected, weighed, and used to calculate the BBIX and bursa-body ratio. The BBIX in the inoculated group was lower than 0.7 after 5 and 7 days of infection. At 14 days post-infection, the BBIX was higher than 0.7 (Figure 2B), suggesting that the damage of BF tissues was gradually restored. Meanwhile, bursa-body ratio of challenged chickens was significantly reduced at the beginning of 3 days of infection in comparison to that in control chickens (Figure 2C). Figure 2D depicts that BF tissues exhibited obvious pathological damage at 3 days post-infection with SM01. The lymphocytes in the dermis and medulla necrotic cells were necrotic, dissolved, and significantly reduced, and there was a large proliferation of epithelial cells and connective tissue. In comparison with the challenged group chickens, no obvious pathological damage was observed in the chickens of the control group.

In conclusion, this study demonstrated the isolation of a vvIBDV, with both segments A and B derived from a vv strain.

## Data Availability

The original contributions presented in the study are included in the article/supplementary material, further inquiries can be directed to the corresponding author.
